# Pharmacokinetics of Curative Tranexamic Acid in Parturients Undergoing Cesarean Delivery

**DOI:** 10.3390/pharmaceutics14030578

**Published:** 2022-03-06

**Authors:** Sixtine Gilliot, Anne-Sophie Ducloy-Bouthors, Florence Loingeville, Benjamin Hennart, Delphine Allorge, Gilles Lebuffe, Pascal Odou

**Affiliations:** 1 ULR-7365-Groupe de Recherche sur les Formes Injectables et les Technologies Associées (GRITA), Université de Lille, Centre Hospitalier Universitaire de Lille, F-59000 Lille, France; anne-sophie.bouthors@chru-lille.fr (A.-S.D.-B.); gilles.lebuffe@chru-lille.fr (G.L.); pascal.odou@univ-lille.fr (P.O.); 2 Institut de Pharmacie, Centre Hospitalier Universitaire de Lille, F-59000 Lille, France; 3 Pôle Anesthésie-Réanimation, Hôpital Huriez, Centre Hospitalier Universitaire de Lille, F-59000 Lille, France; 4 ULR2694—METRICS—Evaluation des Technologies de Santé et des Pratiques Médicales, Université de Lille, Centre Hospitalier Universitaire de Lille, F-59000 Lille, France; florence.loingeville@univ-lille.fr; 5 Unité Fonctionnelle de Toxicologie, Pôle Biologie Pathologie Génétique, Centre Biologie Pathologie, Centre Hospitalier Universitaire de Lille, F-59000 Lille, France; benjamin.hennart@chru-lille.fr (B.H.); delphine.allorge@chru-lille.fr (D.A.); 6 ULR 4483-IMPECS-IMPact de l’Environnement Chimique sur la Santé, Faculté de Médecine-Pôle Recherche, Université de Lille, 1 Place de Verdun, F-59045 Lille, France

**Keywords:** caesarean section, intravenous, pharmacokinetics, postpartum hemorrhage, tranexamic acid

## Abstract

The aim of this study was to evaluate the population pharmacokinetics of tranexamic acid (TXA) administered intravenously at a single dose of 0.5 or 1 g in parturients undergoing active hemorrhagic cesarean delivery and to evaluate the influence of patient variables on TXA pharmacokinetics. Subjects from three recruiting centers were included in this PK sub-study if randomized in the experimental group (i.v TXA 0.5 g or 1 g over one minute) of the TRACES study. Blood samples and two urinary samples were collected within 6 h after TXA injection. Parametric non-linear mixed-effect modeling (Monolix v2020R1) was computed. The final covariate model building used 315 blood and 117 urinary concentrations from seventy-nine patients. A two-compartment model with a double first-order elimination from the central compartment best described the data. The population estimates of clearance (CL), central volume of distribution (V1), and half-life for a typical 70 kg patient with an estimated renal clearance of 150 mL/min (Cockroft–Gault) were 0.14 L/h, 9.25 L, and 1.8 h. A correlation between estimated creatinine clearance and CL, body weight before pregnancy, and V1 was found and partly explained the PK variability. The final model was internally validated using a 500-run bootstrap. The first population pharmacokinetic model of TXA in active hemorrhagic caesarean section was successfully developed and internally validated.

## 1. Introduction

Post-partum hemorrhage remains the first direct obstetrical cause of worldwide maternal morbidity. It is defined as a blood loss of more than 500 mL after vaginal birth or 1000 mL after caesarean section within 24 h of giving birth [1]. Even if the maternal mortality ratio has fallen by 45% between 1990 and 2013, it remains above the expectations, especially in developing countries [2,3].

To reduce the incidence of PPH, the WHO and professional bodies recommend active management of PPH using prophylactic administration of uterotonics in addition to other non-pharmacological interventions. In 2012, the WHO issued guidelines for administrating intravenously tranexamic acid when uterotonics are unavailable or where the bleeding may be partly due to genital tract trauma [4]. Tranexamic acid (TXA) is an antifibrinolytic drug that was found to reduce bleeding by inhibiting the breakdown of fibrin and fibrinogen by plasmin [5]. The treatment regimen proposed by the WHO consists of an early dose of 1 g of TXA, over 10 min, followed by a second dose of 1 g if bleeding continues after 30 min or restarts within 24 h of completing the first dose [4]. This treatment regimen was defined according to two major studies, the EXADELI and WOMAN trials, whose treatment regimen was itself derived from studies conducted on trauma patients (notably the CRASH-2 trial) [6,7,8].

In healthy volunteers, the PK of TXA is well described; TXA is reported to be eliminated by urinary excretion via glomerular filtration with more than 95% of the dose excreted unchanged in healthy volunteers. Accordingly, only a small fraction of TXA acid is assumed to be metabolized by the liver [9]. The complexity of pharmacokinetics (PK) in hemorrhagic caesarean delivery is related to the alterations of body mass and elimination of drugs due to pregnancy-induced physiological changes (increased plasma volume and renal clearance, anemia, and hypercoagulability) and surgery-induced changes (blood loss and reduced blood mass, inflammation, and oliguria) [10]. Thus, the drug disposition reported in healthy patients [9] and trauma patients [8] may not be applicable to the drug disposition in parturients undergoing hemorrhagic caesarean section (CS).

Except for our preliminary model out of the TRACES pilot sub-study [11], a literature search failed to identify studies specifically addressing the hemorrhagic population for the TXA PK model during CS. The TRACES pilot study aimed to construct the PK model of tranexamic acid in patients receiving a single 0.5, 1 or 2 g intravenous (i.v) bolus and to identify the factors most closely tied up to therapeutic variability between individuals. The results suggested a partial non-urinary elimination of TXA in this population. However, the sample of patients included in the study was small and validation of the findings on a larger cohort is needed.

The current study was conducted with the following aims: (i) to characterize the PK of TXA administered intravenously at a single dose of 0.5 or 1 g over one minute in parturients undergoing hemorrhagic CS; (ii) to assess the hypothesis of a partial non-urinary TXA elimination; and (iii) to evaluate the influence of patient variables on TXA PK.

## 2. Materials and Methods

### 2.1. Ethics Approval and Consent to Participate

The TRACES trial was conducted in accordance with the Article L. 1121-4 of the French Public Health Code. Approvals were obtained from the Regional Ethics Committee (15/50_020216) and the Competent Authority in France (ANSM 201500249926). It was registered and assigned the NCT number NCT02797119.

The consent procedures were described in detail in the TRACES trial protocol [12].

### 2.2. Patients and Data Collection

TRACES trial participants recruited in the Lille, Paris Louis Mourier, and Paris Trousseau centers were considered for inclusion. Each patient admitted for CS before or during labor was informed before the beginning of the CS; a signed consent was collected. Patients were included when postpartum hemorrhage was superior to 800 mL according to the TRACES trial protocol [12]. The choice of the bleeding volume cut-off was chosen according to our current practices. The time-point before injection was named T0. A single 10-mL vial containing 0, 0.5, or 1 g of TXA (Exacyl^®^ 0.5 g/5 mL, Sanofi-Aventis, Compiègne, France) was administered blindly over 1 min in included patients. The intravenous injection was performed using a strict control of 1 min duration. T1 was defined the time-point of the end of injection. It was suggested that T1 corresponded to the plasma concentration peak of TXA. A rescue second dose of 0.5 g or 1 g was given if hemorrhage became severe (total blood loss >1500 mL). Blood loss volume (mL) was measured using the surgical or cell saver aspiration bag, the under-buttock delivery bag collecting vaginal blood flow during CS, and by weighing drapes and pads.

### 2.3. Measurements and Data Handling

Age, body weight, height, serum creatinine concentrations, and plasma urea concentrations were collected for each included patient at inclusion time.

Maternal blood samples were collected from the opposite arm to that of drug administration through a peripheral venous catheter. The venous blood samples were taken in citrated and EDTA tubes of 4 mL each at 15, 30, 60, 120, 180, and 360 min (defined as T15, T30, T60, T120, T180, and T360) +/− 10 min after the injection according to the study protocol. The urinary samples were collected within 6 h after treatment. The exact time of blood and urine sampling were collected to perform reliable PK modeling.

The differences between the baseline characteristics of the experimental groups (TXA0.5g and TXA1g) were tested according to the results of the homogeneity of variance tests. If the assumption of homogeneous variances was met, an independent-samples *t*-test (α = 5%) was performed. If the assumption of homogeneous variances was rejected, a Kruskal-Wallis test (α = 5%) was conducted.

### 2.4. Sample Analysis

Blood and urinary samples were analyzed by the toxicology laboratory of the Lille Hospital Centre according to the protocols displayed in the TRACES trial protocol.

Four hundred microliters of methanol containing 7β-hydroxyethyl-theophylline (Sigma-Aldrich, Saint Quentin Fallavier, France) at 20 mg·L^−1^ were added to a 50 µL-sample of plasma or diluted urine (1:10). This mixture was centrifuged (4500× *g*, 4 °C, 10 g). Water/formic acid at a concentration of 0.1% (180 μL) was mixed with 20 µL of the obtained supernatant.

A liquid chromatography system coupled with tandem mass spectrometry (Acquity Xevo-TQ Detector, Waters, Milford, MA, USA) was used to achieve the dosages written in the protocol [13]. The system was equipped with an HSS T3 column (1.8 μm × 2.1 × 50 mm) maintained at +50 °C. The mobile phase gradient consisted of methanol and formic acid. A positive ion mode, using multiple reaction monitoring (MRM), was used to detect each ion of the separated compound. A 5 µL volume was used for the injection of all the analyses. Data acquisition and quantification were performed using MassLynx 4.1 Software (Waters).

Concerning the qualification data for the HPLC-MS/MS detection assay, performed within the range of 5–200 mg/L, the correlation coefficient was 0.995. The repeatability and the inter-day precision obtained were, respectively, <2.90% and 4.15% for a 150 mg/L-spiked sample, and <3.80% and 5.30% for a 20 mg/L-spiked sample.

### 2.5. PK Modeling

The PK model was developed sequentially using non-linear mixed-effect modeling software (Monolix version 2020R1, Lixoft, Antony, France). Parameters were estimated by computing the maximum likelihood estimator using the stochastic approximation expectation-maximization (SAEM) algorithm combined with a Markov Chain Monte Carlo (MCMC: 5 for the number of chains) procedure.

**Figure 1:**
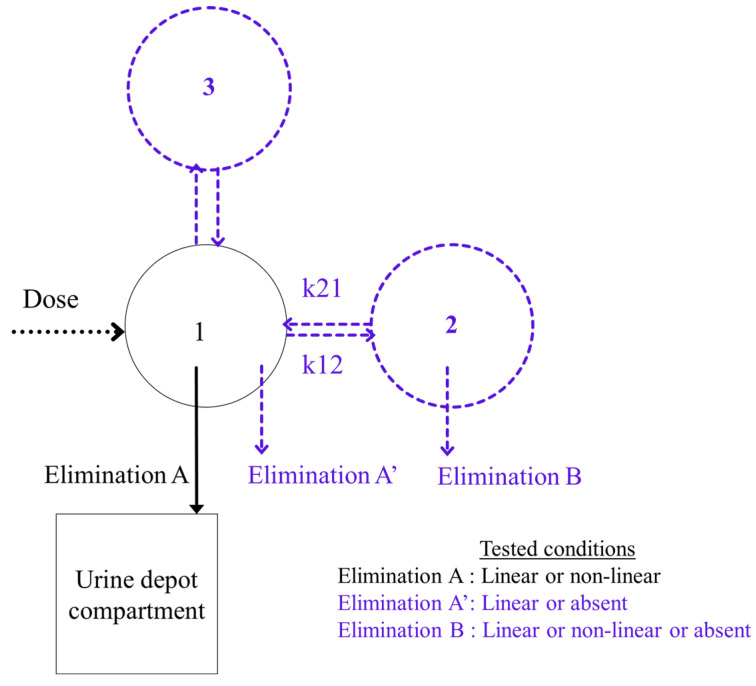
Hypothetical base models tested. The hypothetical tested parameters are represented in blue color.

In this article, non-linear mixed-effect models (NLMEM) were considered to model y_i,j_, the plasma or urinary concentration in TA estimated for a patient i (i = …) at time j (j = …) as follows:

(1) $$y_{i,j}=f\left(t_{i,j},\psi_{i}\right)+g\left(t_{i,j},{\;\psi }_{i}\right){*\varepsilon }_{i,j},\;\varepsilon_{i,j}\;\sim \;N\;(0,\;1)$$

where f represents the nonlinear function of the model, ψ_i_ represents the vector of an individual PK parameter for subject i, g represents the residual error model, and ε_i,j_ represents the residual error. As we worked on parametric software, the normality of ε_i,j_ was assumed. The random variation in the population PK parameters was described by between-subject variability (BSV) for every fixed-effect assuming that parameters were log-normally distributed. The tested base models are displayed in Figure 1.

Compartment 1 represents the central compartment, while compartments 2 and 3 represent hypothetical peripheral compartments (Figure 1). The rationale to test a potential elimination from compartment 2 comes from the possibility that it may represent the uterine hemorrhagic compartment. In that case, the elimination of TXA in the hemorrhagic blood would be reflected by elimination from compartment 2. The only elimination that was not hypothetical was the elimination of TXA from compartment 1 as TXA concentrations were measured in the urine within 6 h after TXA administration. The fraction of TXA eliminated via urine is considered as “*p_urine_*”, and is calculated as follows:

$$p_{\mathrm{urine}\;}=k_{\mathrm{urine}\;}\times \frac{V1}{CL}\;and\;k_{\mathrm{urine}\;}=\frac{dA_{u}}{dt}\times \frac{1}{A_{1}}$$

where $A_{1}$ is the amount of TXA in the central compartment, and $A_{u}\;$is the amount of TXA in the urine compartment.

Additive, proportional, combined (additive and proportional), and exponential error models were assessed to model the residual unexplained variability.

The choice of the base and error models was based on the calculation of the objective function (OFV) using the corrected Bayesian Information Criterion (BICc), which was penalized from the maximized log-likelihood by a term that depended on the number of fixed effect parameters and the sample size. A model X was better than a model Y if it led to a reduction in the BICc of at least 3.84 points (value taken from χ²ndl = 1 distribution at α = 0.05) compared to model Y. Furthermore, a low value of the condition number κ (i.e., <100) suggested that the model was not over-parameterized and that there was an absence of collinearity between the PK parameters.

Once the best base model was selected, the influences of covariates were tested. We evaluated the covariates of age, body weight (BW), body mass index (BMI), lean body weight (LBW), adjusted body weight (ABW), ideal body weight (IW), body surface area (BSA), and estimated creatinine clearance (eClcr) using the Cockcroft–Gault formula-estimated Glomerular Filtration Rate (eGFR) based on the CKD-EPI formula or the MDRD formula (GFR_MDRD). The formulas used to calculate each parameter are displayed in Appendix A, Table A1. Each characteristic (BMI, LBW, IW, BSA, eClcr, and eGFR) was calculated twice, first using the body weight measured before pregnancy and secondly using the body weight measured after pregnancy. The choice of testing the body weight measured before pregnancy as a potential covariate was related to the fact that the weight gained during pregnancy does not correspond to a proportional increase in fat tissues in the body. Accordingly, the body weight measured before pregnancy could be a better estimator of the distribution than the body weight measured on the day of CS. The effect of covariates was tested on each PK parameter, one at a time, by incorporation into the base model by the following relationship using a forward selection:

(2) $$\log\left(\psi_{i,\ell }\right)=\log\;(\theta_{\ell })+{\;\beta }_{1}{*x}_{1}+{\;\eta }_{i,\ell },\;\eta_{i,\ell }\;\sim \;N\;(0,{\;\omega }_{\ell })$$

where $\left(\psi_{i,\ell }\right)$ represents the ℓth individual PK parameter (ℓ = (1, …, P)), in which P is the total number of PK parameters. $\theta_{\ell }$ represents the fixed effect of the PK parameter ℓ, $\beta_{1}$ represents the effect of the covariate $x_{1}$, $\eta_{i,\ell }$ represents the ℓth element of the vector $\eta_{i}$, capturing the BSV term on parameter ℓ for subject i, and $\omega_{\ell }$² represents the variance of the interindividual error.

Women for whom weight and serum creatinine concentration were missing were excluded from the covariate analysis.

The selection of the covariates was computed in the same way as the selection of the base and error model. A covariate was retained if it significantly explained the BSV of a PK parameter (Wald test, *p* ≤ 0.05).

The performance evaluation of the final covariate model used the precision of the parameter estimation expressed as the relative standard error (RSE, in %), which was required to be ≤30% for fixed parameters and ≤50% for $\omega_{\ell }$; and diagnostic plots (1–3), based on Monte Carlo simulations. (1) The Visual Predictive Check (VPC) plot assessed whether the model could reproduce the variability in the observed data from which it originated by overlapping the distribution of observations and the distribution of predictions. (2) Scatter plots of observed and predicted values graphically compared the distance between the observed and predicted value, which was expected to be as low as possible. (3) A histogram of the distribution of the Normalized Prediction Error (NPDE) was computed and expected to follow the N(0, 1) distribution. A Shapiro-Wilk test (α = 5%) was computed to assess the normality of the random effects $\eta_{i,\ell }$ and $\varepsilon_{i,j}$.

Model performance evaluation graphs were exported from Monolix v2020R1 computations.

### 2.6. Noncompartmental Analysis

Noncompartmental PK analysis (NCA) was performed using PKAnalix (version 2020R1, Lixoft) to estimate the area under the curve (AUC) from T1 to T30 (AUC T1-T30) and the AUC from T1 to T60 (AUC T1-T60), to predict the mean residence time (MRT) of TXA for each patient and the maximal blood concentration (C_T1_). The integral method was computed using linear log trapezoidal parametrization.

Groups of patients were formed according to the bleeding status of patients at T30 and T60. For the analysis performed at T30, group A consisted of the parturients who had definitely stopped bleeding from T30, group B corresponded to the patients who had continued to bleed, and in group C were gathered the patients who had stopped bleeding at T30 but had bled again at T60, T120, or T360.

For the analysis performed at T60, group A consisted of the parturients who had definitely stopped bleeding from T60, group B corresponded to the parturients who continued to bleed at T60, in group C were gathered the patients who had stopped bleeding at T60 but had bled again at T120 or T360, and finally, group D consisted of the patients who stopped bleeding between T0 and T30 or between T30 and T60 and who bled again at T60.

Comparisons of the mean values for C_T1_, TXA blood concentrations measured at T30 (C_T30_), AUC(T1-30), and MRT were computed between the different groups according to the bleeding status at T30.

Comparisons of the mean values for C_T1_, TXA blood concentrations measured at T60 (C_T60_), and AUC(T1-60) were computed between the different groups according to the bleeding status at T60.

Comparisons were computed using a Kruskal-Wallis test with an alpha-risk of 5%.

### 2.7. Model Internal Evaluation

The robustness of the model was assessed by performing a 500-run bootstrap resampling procedure in Monolix using the Rsmlx package (version 2.0.2) in R software (version 3.6.1). The median values obtained from the 500 bootstrap runs were discussed regarding the mode values of the estimated values of fixed parameters. Final PK parameters were re-estimated for 500 samples and the median, first quartile (Q1), and third quartile (Q3) were calculated for each parameter. The mode values obtained from the original dataset were expected to be close to the median values obtained from the bootstrap runs and within the range of (Q1;Q3). The validation of a non-linear mixed-effect model was underpinned by the precision of the estimations obtained from the bootstrap, which mostly needed to reach the required standards (≤30% for fixed effect parameters and ≤50% for $\omega_{\ell }$).

### 2.8. Simulations to Derive Optimal Dosing

Monte Carlo simulations of 1000 parturients were repeated for the two-dose regimens, 0.5 and 1g, given as single bolus doses. Results were computed using the mlxR package (version 4.0.6) and the graphic presentation of the outputs was computed with the ggplot2 package (version 3.3.3) in R software.

## 3. Results

### 3.1. Recruitment

**Figure 2:**
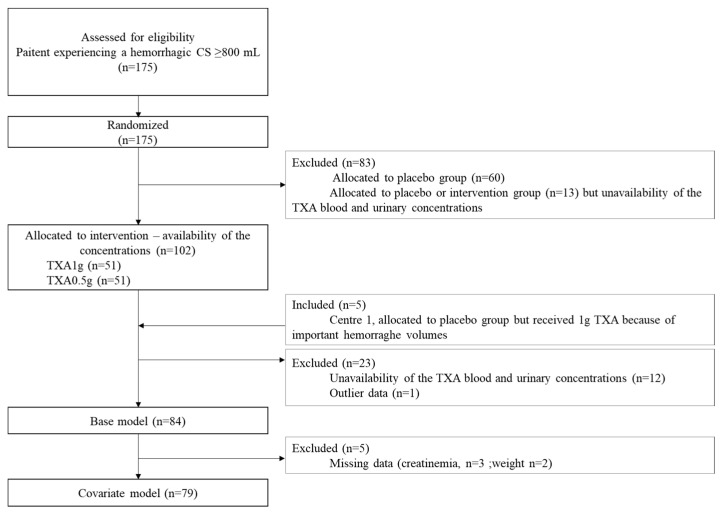
Study flowchart. Legend: CS, caesarean section; TXA, tranexamic acid.

In total, 175 hemorrhagic patients were enrolled in the TRACES study. Eighty-four patients were recruited in the TRACES PK sub-study (Figure 2).

The patients’ baseline characteristics are displayed in Table 1.

Baseline characteristics suggest that the two regimen groups are well-balanced. Rescue doses were administered in seven patients in the TXA1g group (1 g once for six of them and 1 g twice for one of them) and one patient in the TXA0.5g group (0.5 g once). These eight patients were included in the populational PK modeling, and their therapeutic scheme was rigorously codified in the dataset.

### 3.2. Exploratory Analysis

A total of 335 blood and 154 urine concentration points obtained from the eighty-four included patients were computed to build the base model. Furthermore, 315 blood and 147 urine concentration points obtained from seventy-nine patients were used to build the final covariate model. The minimal and maximal concentrations measured at T15 were 11 and 38 mg/L after a single 0.5 g dose, and 36.9 and 67.8 mg/L after a single 1 g dose. Concentrations declined rapidly for the first hour, followed by a slighter decline until 360 min, at which time more than 90% of TXA was eliminated for half of the patients. The biphasic profile suggested a two-compartment disposition of TXA. Half-life was calculated at 111 min (95;127) (median (Q1;Q3) according to non-compartmental analysis computed on the base model.

Concerning the eight participants who had received a rescue dose, a table summarizing the individual baseline data and the individual estimated PK parameters is presented in Appendix C.

### 3.3. Base Model

The results of the base model building are presented in Table 2.

**Figure 3:**
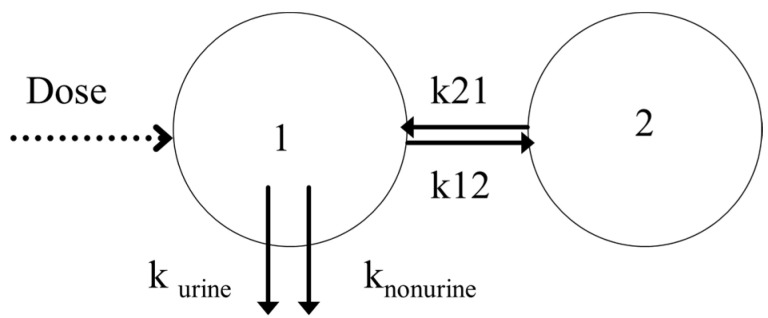
Base model result: two-compartment model with a double linear elimination (model A).

The base model that fitted best with the data was the same as the one chosen in our preliminary study (Figure 3).

The chosen model was a two-compartment model with a double first-order elimination from the central compartment. This base model was characterized by an elimination clearance (CL), a volume of central (V1) and peripheral (V2) compartments, a diffusional clearance (Q), and a urinary excretion fraction (purine). The estimate values of the PK parameter for this base model were 0.14 L/min for CL, 17.66 L for V1, 0.079 L/min for Q, 8.12 L for V2, and 0.61 for purine.

The combined error model was the most adequate for evaluating the interpatient and residual variability of the blood concentrations. This model is written as follows:

(3) $$g\left(t_{j},\psi_{i}\right)=\sqrt{a1²+b1²\times f\left(t_{j},\psi_{i}²\right)}$$

where f represents the nonlinear function of the model, g represents the residual error of the model, and a1 and b1 represent the fixed factors of the combined residual error for the blood concentration. The proportional model was the most adequate for evaluating the interpatient and residual variability of the urine concentrations. This model is written as follows:

(4) $$g\left(t_{j},\psi_{i}\right)=b2\times f\left(t_{j},\psi_{i}\right)$$

where b2 represents the fixed factors of the proportional residual error for the urine concentrations.

### 3.4. Covariate Model

The results of covariate testing showed that anthropometric parameters significantly affected the central volume of distribution of TXA with a risk of 5% bilateral with body weight measured at the beginning (BWbef) and the end of pregnancy (BW) affecting the BSV more significantly than the other parameters (r^2^ = 0.41, *p*-value = 1.87 × 10^−4^).

It was also found that CL was affected by renal parameters (estimated creatinine clearance (eClcr) determined using the CG formula calculated based on BWbef (r^2^ = 0.65, *p*-value = 1.09 × 10^−10^); estimated Glomerular Filtration Rate (eGFR) determined using the CKD-EPI formula calculated based on BWbef (r^2^ = 0.61, *p*-value = 3.47 × 10^−9^)).

According to the results (Table 3), the model with the lowest BICc was the one for which eClcr determined using the CG formula calculated based on the BWbef was added as a covariate of CL and BWbef was added as a covariate of V1.

This model was considered as the final covariate model (model C). Model D was not chosen because of over-parametrization (κ > 100).

According to Table 3, TXA CL and V1 for the patient *i* were described as follows:

$$CL_{i}=CL_{pop}\times e^{\beta CL\times eClcr}\;and\;V1_{i}=V_{pop}\times {\left(\frac{BW_{i}}{70}\right)}^{\beta V1\;}$$

The final estimated PK parameters identified for model C are summarized in Table 4.

The estimated PK parameters were close to the median of the 500-run bootstrap estimates and within the range of (Q1;Q3) obtained from bootstrap, suggesting the robustness of the final PK model. Shrinkage values were found to be low, which suggests a good estimation of the individual parameters and attests that the diagnostic graphs are interpretable.

**Figure 4:**
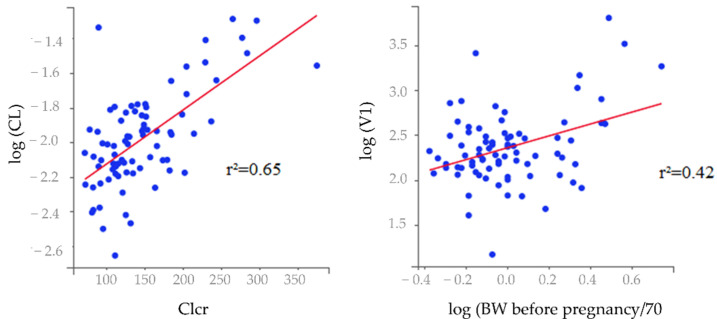
Correlations between the conditional mean of individual pharmacokinetic parameters and covariates of the final covariate model. Individual parameters are displayed by blue dots and regression line is displayed by the red line.

Correlations between log(BWbef/70) and log(V1), and log(ClCr) and log(CL) are represented in Figure 4.

The significance of the Wald test was consistent with keeping the eClcr as a covariate for CL (*p* < 2.2 × 10^−16^) and log(BWbef/70) as a covariate for V1 (*p*-value = 1.04 × 10^−4^).

**Figure 5:**
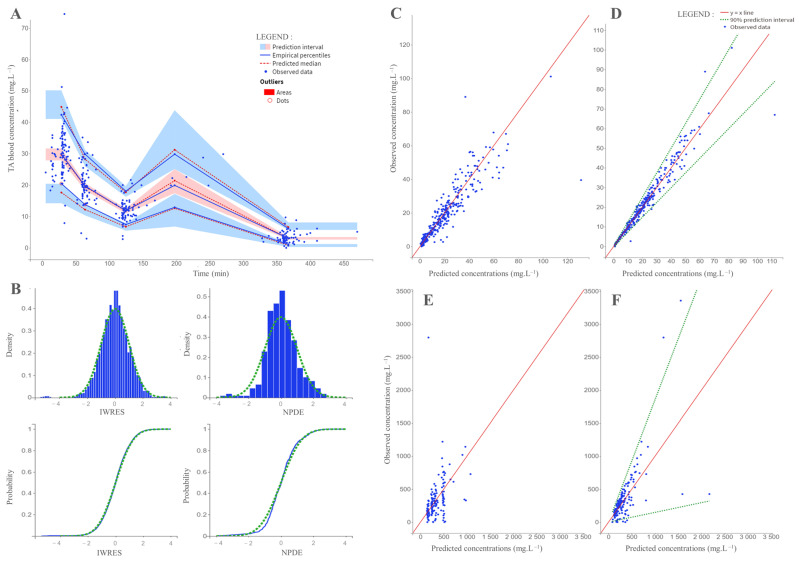
Goodness-of-fit plots obtained from the final covariate model: (**A**) The visual predictive check graph represents the observed plasma concentrations in tranexamic acid (TXA) plotted against time, based on 1000 Monte Carlo simulations. Prediction intervals for each percentile are estimated across all simulated data and represented as colored areas (pink for the 50th percentile, blue for the 10th and 90th percentiles). Observed data are displayed as blue dots. Predicted medians and empirical percentiles are displayed as green dotted lines and blue lines, respectively. (**B**) Normalized prediction distribution errors (NPDE) plotted against time and tranexamic acid (TXA) plasma concentrations. Observations plotted against populational (**C**) and individual (**D**) predicted tranexamic acid (TXA) plasma concentrations. Observations plotted against populational (**E**) and individual (**F**) predicted tranexamic acid (TXA) urinary concentrations. The limits of the 90% confidence intervals are displayed as green dotted lines. Concerning the VPC graph, curves representing empirical percentiles overlaid the prediction intervals. Concerning the observations versus predictions graph, the ratio of observed/predicted concentrations was around the x = y line for both plasma and urinary values. The proportions of plasma and urinary ratios that fell outside the 90% prediction interval were 2.2% and 6.8%, respectively. The distribution of NPDE was quite well adjusted to the density of the standard Gaussian distribution. The Shapiro–Wilk test of normality was significant, but significance is often observed when analyzing a large number of observations (Fc = 0.98, *p*-value = 5.8 × 10^−5^ for blood concentrations; Fc = 0.96, *p*-value = 4.84 × 10^−4^ for urinary concentrations) [14].

Diagnostic plots of the final covariate model are presented in Figure 5.

**Figure A1:**
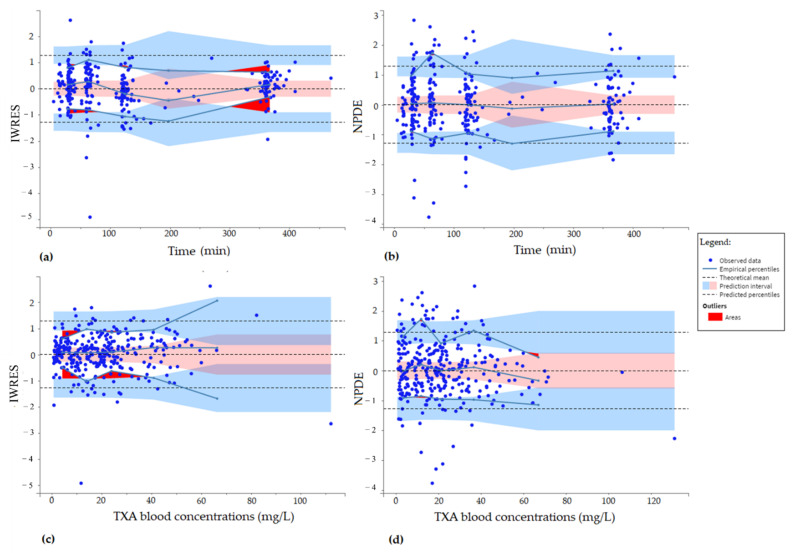
Individual weighted residuals (IWRES) plotted versus time (**a**) and tranexamic acid (TXA) blood concentrations. (**c**) Normalized prediction distribution errors (NPDE) plotted versus time (**b**) and TXA blood concentrations (**d**).

Apart from a few outliers, the NPDE plots did not suggest any misspecification of the model (Appendix B, Figure A1). The normal distribution of the η values was not rejected by the Shapiro-Wilk test at 5% except purine (Fc = 0.91, *p* > 8.02 × 10^−6^).

In conclusion, TXA CL and V1 for the patient *i* were described as follows:

$$CL_{i}=0.077\times e^{0.0039\times eClcr}and\;V1_{i}=9.25\times {\left(\frac{BW_{i}}{70}\right)}^{1.41\;}$$

### 3.5. Simulations

**Figure 6:**
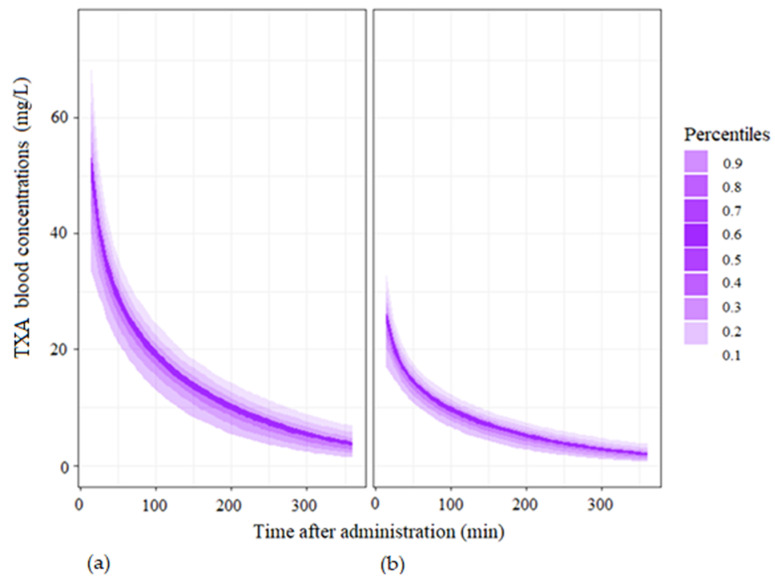
Monte Carlo simulations with the base model for 1000 individuals. Legend: (**a**) administration of a single dose of 1 g of tranexamic acid at T0; (**b**) administration of a single dose of 0.5 g of tranexamic acid at T0.

Monte Carlo simulations of 1000 parturients receiving single doses of 0.5 and 1 g are displayed in Figure 6.

Our results suggest that 90% of individuals maintained a blood concentration ≥30 mg/L and ≥15 mg/L for the first 15 min following the administration of a 1 g or 0.5 g bolus of TXA, respectively. The mean concentrations estimated from the Monte Carlo simulations are presented in Table 5.

Rescue doses were administered at a median time of 87 min after the first administration, with two patients requiring a rescue dose 35 min after the first administration and one patient requiring a rescue dose at T50 min.

### 3.6. Noncompartmental Analysis

**Figure 7:**
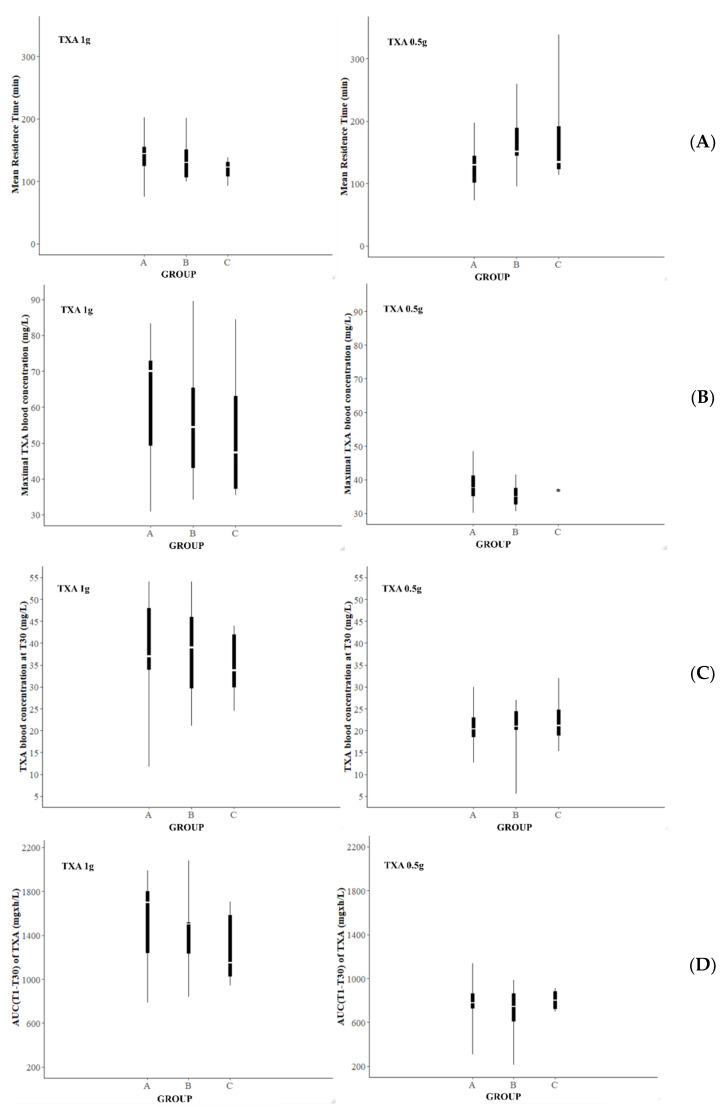
Representation of the mean resident time of TXA (**A**), the predicted maximal blood concentration of TXA (**B**), the blood concentration of tranexamic acid measured 30 min after the TXA administration (**C**), and the AUC estimated over the 30 min following the TXA administration (**D**) according to the groups of bleeding status defined 30 min after the first TXA injection. Legend: AUC, area under the curve; TXA, tranexamic acid.

**Figure 8:**
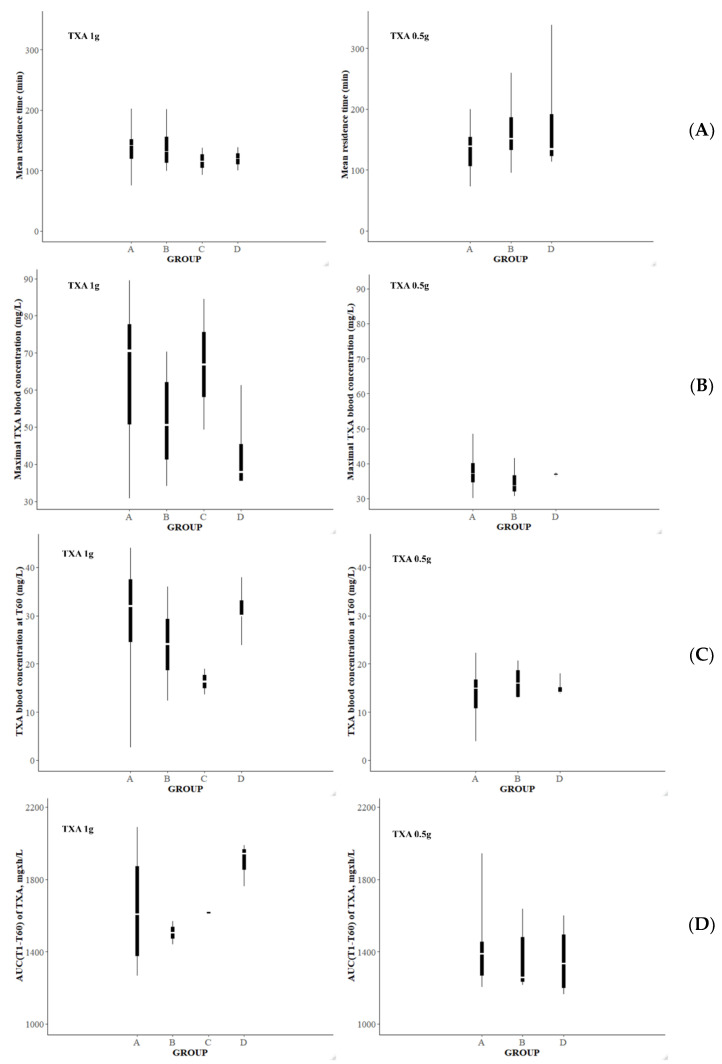
Representation of the mean resident time of TXA (**A**), the predicted maximal blood concentration of TXA (**B**), the blood concentration of tranexamic acid measured 60 min after the TXA administration (**C**), and the AUC estimated over the 60 min following the TXA administration (**D**) according to the groups of bleeding status defined 60 min after the first TXA injection. Legend: AUC, area under the curve; TXA, tranexamic acid.

Results of the NCA are presented in Figure 7 for the analysis performed according to bleeding status at T30 and Figure 8 for the analysis performed according to bleeding status at T60. Missing data correspond to data for which the number of concentrations collected could not provide a correct analysis.

Our results showed no significant differences for C_T1_, C_T30_, AUC (T1-T30), and MRT between the groups defined according to their bleeding status at T30. Our results also failed to point out any significant differences for C_T1_, C_T60_, MRT, and AUC (T1-T60) between the groups defined according to their bleeding status at T60.

However, the boxplots suggest that the AUC (T1-T30) and the AUC (T1-T60) were likely to be higher in the group of patients for whom there was a definitive cessation of bleeding (group A) than in the other groups of patients (group B and C; and group D for T60 analysis), exclusively for patients recruited in the TXA1g group. The same observation could be made for the predicted maximal concentration at T1 (C_T1_). Concerning the results for patients recruited in the TXA0.5g group, C_T1_, AUC (T1-T30), and AUC (T1-T60) were found to be quite similar regarding the status of bleeding of the patients. Concerning the MRT, C_T30_, and C_T60_ values, no tendency was noticed for both groups (TXA0.5g and TXA1g).

Concerning the eight participants who received a rescue dose of TXA, the anthropometric and biological baseline values and the individual PK parameters are summarized in Appendix C. No noticeable value was noted in their baseline characteristics or their individual estimated PK parameters.

## 4. Discussion

TRACES is the first population PK study of hemorrhagic parturients receiving intravenous TXA during caesarean section. TXA PK data were adequately described by a two-compartment model. PK modeling highlighted that BSV was partly explained by renal clearance estimated using the Cockroft-Gault formula and body weight normalized to a 70 kg individual measured before pregnancy. The PK results showed that the predicted TXA blood concentrations rapidly declined below 20 mg/L, around 30 min after the administration of a single bolus dose of 0.5 g. The urinary elimination of TXA considered in the study design and the large number of patients recruited contributed to the robustness of the model.

In healthy volunteers, trauma patients, and patients undergoing cardiopulmonary bypass, the TXA PK data were also best described by a two-compartment model [15,16,17,18,19], with TXA clearance between 6.6 and 10.1 L/h and the TXA central volume of distribution between 4.8 and 17.9 L, both normalized to the body weight of a 70 kg-individual. More recently, Li et al. [20] conducted a populational PK study on 30 patients undergoing elective caesarean surgery. In this study, TXA was administered intravenously at prophylactic doses of 5 mg/kg (n = 10), 10 mg/kg (n = 10), and 15 mg/kg (n = 10) at the time of umbilical cord clamping. Their results also suggested that TXA PK was best described by a two-compartment model with first-order elimination. TXA clearance was estimated at 9.4 L/h, and the central volume of distribution was estimated at 10.1 L.

In our study, the estimated values for the PK parameters found were from 8.4 L/h for clearance normalized for creatinine clearance to 150 mL/min calculated using the Cockroft–Gault equation, and 9.25 L·70 kg^−1^, normalized based on a body weight of 70 kg. Both results are consistent with the findings of Li et al. [20] and with our preliminary results, suggesting an estimated populational clearance and central distribution volume of 10.3 L/h and 11.5 L, respectively [11]. One of the parameters that varied significantly in comparison with the preliminary model estimations was the fraction of urinary elimination. It was estimated at around 50% in this study versus 25% in the previous study. This variability may be associated with a covariate that has not been investigated yet; it will be interesting to study the variability of the purine regarding the pharmacodynamics to investigate whether there may be any balance between the renal elimination and the TXA efficacy. Indeed, the mechanism of action of TXA consists of inhibiting fibrinolysis by preventing the plasminogen and t-PA from binding to fibrin. An assumption of the non-urinary elimination of TXA could be the trapping of TXA between plasmin and fibrin according to the mechanism of action of TXA that would influence either the efficacy of TXA or the fraction of TXA excreted in the urine. The second assumption that could explain the non-urinary elimination of TXA is the elimination of TXA through the uterine hemorrhagic blood flow. Unpublished results revealed that a part of the TXA is eliminated through the hemorrhagic blood flow; however, imprecisions concerning the collection of that blood made it difficult to determine the real concentration of TXA in the hemorrhagic blood and led us to exclude those blood uterine concentrations from the PK analysis. In fact, the collecting process was considered too imprecise to measure the exact volume of uterine blood and, thus, the excreted amount of TXA. The precision of the uterine blood collection was considered as less important than the management of the hemorrhagic CS in our study.

Our results are also consistent with our preliminary results that suggested a partial urinary elimination of TXA [11]. The renal excretion of TXA is in accordance with the renal clearance explaining the BSV on urinary clearance of TXA. Previous studies reported that the eClcr and the eGFR of healthy pregnant women were poorly estimated with the existing prediction equations (i.e., Cockcroft-Gault, MDRD, and CKD-EPI) [21,22]. Unexpectedly, the eClcr according to the Cockroft–Gault formula was found to be correlated significatively with the individual CL (r^2^ = 0.65). This is the first study suggesting a correlation between the BSV on CL and the estimated renal clearance according to the Cockroft–Gault formula.

The body weight of individuals reported before pregnancy was found to influence the BSV of V1, as described in previous studies [15,16,17,18].

The implementation of both eClcr and BW in the model provided a reduction in the BICc of 55 points while maintaining an acceptable condition index (below 100).

The incoherence of our final covariate model with our preliminary results is easily explained by the larger number of patients included in this new study, which better depicts the effect of the individual covariates on the BSV of PK parameters [11]. Moreover, the amount of data was strengthened by the addition of a second urinary collection point for this study in comparison with the pilot study (one urinary point). Our study is the only one in the literature to include the TXA urinary dosage in the model building.

Concerning the TXA target concentration, a recent review suggested that TXA concentrations of 10–15 mg/L may be sufficient to inhibit hyperfibrinolysis in vivo [23]. According to the authors, this concentration range should be targeted in PK studies. However, this assumption only relies on studies measuring the effect of TXA concentrations on hyperfibrinolysis in vitro. The in vivo relation between the PK and the pharmacodynamics (PD) of TXA remains unclear. A study suggested that an Imax model best described the relationship between the TXA concentration and the maximum lysis measured using a rotational thromboelastometry (ROTEM) analysis [20]. Considering this PKPD relationship, the authors suggest that a dose of 650 mg is sufficient to prevent hemorrhagic CS assuming 10 mg/L as the PK target and a maximum lysis below 17%. Yet, this study was conducted on patients receiving prophylactic doses of TXA that may prevent hemorrhage and the concentrations needed for hemorrhage to occur once this had already started were not considered. No study has yet determined the TXA target concentration required to stop hyperfibrinolysis after the beginning of this phenomenon in the context of hemorrhagic CS.

According to the results of the NCA, no clear cut-offs concerning a target blood concentration or a target AUC of TXA were noticed. However, our results suggest that the concentrations needed to stop the bleeding are higher than those proposed in recent literature (10 or 20 µg/mL) [20,23]. Indeed, each patient receiving a dose of TXA 0.5 g or 1 g easily reached a TXA blood concentration of 20 µg/mL; nevertheless, the bleeding did not stop for many of them. According to our results, the concentration of tranexamic acid tended to be higher in patients who had stopped bleeding, but that result was only suggested for the analysis performed at T30 and for the patients recruited in the TXA1g group. However, no significant difference for any estimated parameter between the different groups of patients formed according to their bleeding status was pointed out in this study. Another noticeable fact is that only two patients stopped bleeding between T30 and T60, and both belonged to the TXA1g group. Finally, no noticeable value concerning the baseline characteristics or individual estimated PK parameters of the eight participants who had received a rescue dose could be identified.

All these results tend to support a 1 g administration with an early repetition within the first hour if the bleeding does not stop. However, as there are currently no data establishing a threshold of concentration as efficient enough to reduce additional blood loss and inhibit fibrinolysis, the TRACES PK/PD trial, including biological parameters and the results of simultaneous thrombin and plasmin generation assays, will be performed to determine a concentration–response relationship for TXA in the context of caesarean hemorrhage.

## 5. Conclusions

The population pharmacokinetic model of TXA in hemorrhagic CS was developed and internally validated. The BSV of CL was partly explained by eClcr and the BSV of V1 was partly explained by BW measured before pregnancy. This model confirmed the assumption of a partial urinary elimination of tranexamic acid given in the preliminary study. Future pharmacodynamic studies may help to propose the best therapeutic schemes.

## Figures and Tables

**Table 1:** Patients’ baseline characteristics, with results displayed as mean and standard deviation.

Characteristics, Mean (SD ^1^)	Group TXA0.5g ^2^ (n = 34)	Group TXA1g ^2^ (n = 45)	*p*-Value
Age (years)	34 (5)	33 (4)	0.47
Height (cm)	166 (8)	164 (7)	0.19
IW ^3^ (kg)	58.3 (7.0)	56.3 (6.3)	0.19
BW 4 before pregnancy (kg)	73.2 (21.1)	72 (17)	0.46
BW (kg)	84.3 (18.6)	83.0 (15.2)	0.73
BMI ^5^ before pregnancy (kg/m^2^)	26.4 (7.0)	26.6 (6.0)	0.54
BMI (kg/m^2^)	30.4 (6.0)	30.8 (5.1)	0.68
ABW ^6^ before pregnancy (kg) correction factor = 0.4 ^1^	68.7 (10.0)	67.0 (8.3)	0.65
LBW ^7^ before pregnancy (kg)	43.9 (7.4)	43.0 (6.3)	0.65
LBW (kg)	47.8 (6.4)	46.9 (5.4)	0.58
BSA ^8^ before pregnancy (m^2^)	1.8 (0.2)	1.8 (0.2)	0.88
BSA (m^2^)	1.9 (0.2)	1.9 (0.2)	0.54
Serum creatinine concentration (mg/dL)	6.5 (1.5)	6.7 (1.6)	0.25
eClcr ^9^ (Cockroft–Gault, mL/min) with BW before pregnancy	150.5 (64.5)	143.4 (53.2)	0.47
eClcr (Cockroft–Gault, mL/min)	172.3 (62.3)	166.3 (56.1)	0.42
eGFR ^10^ (CKD-EPI, mL/min/1.73 m^2^) with BW before pregnancy	122.5 (20.8)	119.7 (17.2)	0.44
eGFR (CKD-EPI, mL/min/1.73 m^2^)	130.3 (19.1)	127.9 (16.4)	0.42
eGFR (MDRD, mL/min/1.73 m^2^) with BW before pregnancy	117.1 (37.1)	111.9 (35.5)	0.42
eGFR (MDRD, mL/min/1.73 m^2^)	124.5 (37.5)	119.5 (37.0)	0.47
Bleeding volume at inclusion (mL)	1091 (273)	1163 (318)	0.34

^1^ Standard deviation, ^2^ Tranexamic acid, ^3^ Ideal weight, ^4^ Body Weight, ^5^ Body Mass Index, ^6^ Adjusted Body Weight, ^7^ Lean Body Weight, ^8^ Body Surface Area, ^9^ estimated Creatinine Clearance, ^10^ estimated Glomerular Fraction Rate.

**Table 2:** Base model building.

Nb ^1^ of Compartments	Elimination A ^2^	Elimination A′ ^3^	Elimination B ^4^	−2LL ^5^	BICc ^6^	Condition Index
1	First-order	-	-	4283.98	4323.8	3
	First-order	First-order	-	4045.05	4101.69	12
2	Non-linear	-	-	4229.73	4301.42	111
2	Non-linear	-	First-order	NA ^7^	NA	NA
2	Non-linear	-	Non-linear	NA	NA	NA
2	First-order	-	-	4097.00	4158.44	6
2	First-order	-	First-order	4127.8	4199.49	33
2	First-order	-	Non-linear	4071.26	4153.57	84
2	First-order	First-order	-	3980.12	4051.81	81
3	First-order	-	-	3987.84	4080.78	14

^1^ Number, ^2^ urinary elimination from compartment 1, ^3^ non-urinary elimination from compartment 1, ^4^ non-urinary elimination from compartment 2, ^5^ maximized log-likelihood, ^6^ corrected Bayesian information criterion, ^7^ non-applicable (attributed to parameters that failed to be found by the algorithm).

**Table 3:** Steps for pharmacokinetic model building, n = 79.

Model	Parametrization	−2LL ^1^	BICc ^2^	κ ^3^
(A): Base model	CL, V1, V2, Q, purine	3774.45	3845.38	12.98
(B): (A)+ covariate effect	* $\log(\mathrm{CL})=\log(\theta_{\mathrm{CL}})+\beta {{\mathrm{CL}}_{\;}}^{4}\times$ eClcr ^5^ + η_CL_^6^	3725.64	3800.94	41.21
	$\log(V1)=\log(\theta V_{1})+\beta V_{1}\times$ BWbef ^7^ + η_V1_	3760.16	3835.46	948.88
	$\log(Q)=\log(\theta V_{2})+\beta_{Q}\times$ BWbef + ηV_2_	3758.82	3834.12	189.46
	$\log(V1)=\log(\theta V_{1})+\beta V_{1}\times$ log(BWbef/70) + η_V1_	3762.93	3838.23	30.82
(C): (B) * + covariate effect	$\log(V1)=\log(\theta V_{1})+\beta V_{1}\times$ BW ^8^ + η_V1_	3710.77	3790.44	227.97
	$\log(V1)=\log(\theta V_{1})+\beta V_{1}\times$ log(BW/70) + η_V1_	3712.91	3792.58	71.90
	$\log(V1)=\log(\theta V_{1})+\beta V_{1}\times$ BWbef + η_V1_	3714.07	3793.74	137.24
	** $\log(V1)=\log(\theta V_{1})+\beta V_{1}\times$ log(BWbef/70) + η_V1_	3711.10	3790.77	35.14
(D): (C) ** + covariate effect	$\log(\mathrm{CL})=\log(\theta_{\mathrm{CL}})+\beta_{\mathrm{CL}}\times$ Age + η_CL_	3705.86	3789.90	346.30

^1^ maximized log-likelihood, ^2^ corrected Bayesian information criterion, ^3^ condition index, ^4^ factor applied to the covariate, ^5^ creatinine clearance estimated according to the Cockroft-Gault equation, ^6^ between-subject variability, ^7^ Body Weight before pregnancy, ^8^ Body Weight, * the best model B according to BICc and κ, ** the best model C according to BICc and κ.

**Table 4:** Parameter estimates of the final model in parturient women undergoing hemorrhagic caesarean section.

Parametrization		Original Dataset		Bootstrap	
Population Parameters	Covariate Effect	Estimated Values (RSE ^1^, %)	Shrinkage (Conditional Distribution) %	Median	(Q1; Q3) ^2^
θ_CL_ (L/min)	$e^{\left(\beta CL\times eClcr\right)}$	0.077 (7.3)	2.88	0.0785	(0.0746; 0.0825)
β_CL_		0.0039 (11.8)	NA ^4^	0.0038	(0.0035; 0.0042)
θ_V1_ (L)	$\beta V1\times \frac{BW{\;}^{3}}{70}$	9.25 (12.0)	0.857	9.76	(7.78; 12.61)
β_V1_	-	1.41 (25.8)	NA	1.31	(0.93; 1.68)
θ_Q_ (L/min)	-	0.32 (15.3)	−3.74	0.30	(0.24; 0.37)
θ_V2_ (L)	-	9.49 (5.1)	2.64 × 10^−4^	9.58	(8.41; 10.36)
θ_purine_	-	0.54 (7.0)	−1.37	0.55	(0.53; 0.57)
ω_CL_ (%)	-	20 (11.2)	-	0.19	(0.18; 0.21)
ω_V1_ (%)	-	59 (14.1)	-	0.48	(0.32; 0.61)
ω_Q_ (%)	-	67 (18.8)	-	0.65	(0.54; 0.74)
ω_V2_ (%)	-	13 (31.9)	-	0.20	(0.14; 0.30)
ω_purine_ (%)	-	46 (11.9)	-	0.44	(0.36; 0.52)
a1	NA	0.44 (33.0)	NA	0.38	(0.10; 0.52)
b1	NA	0.15 (6.8)	NA	0.15	(0.14; 0.16)
b2	NA	0.52 (7.9)	NA	0.52	(0.49; 0.54)

^1^ Residual standard error, ^2^ (first quartile;third quartile), ^3^ body weight before pregnancy, ^4^ non-applicable.

**Table 5:** Estimated mean concentrations after administration of a single dose of 1 g of tranexamic acid and a single dose of 0.5 g of tranexamic acid.

Mean (10th–90th Percentile)	15 min after Administration	30 min after Administration	60 min after Administration	120 min after Administration	360 min after Administration
Single dose of 0.5 g of TXA	55.1 (35.3;77.8)	39.2 (28.7;51.6)	27.5 (19.6;35.6)	17.3 (10.7;23.9)	4.6 (1.3;8.8)
Single dose of 1 g of TXA	27.1 (17.3;37.6)	19.6 (13.9;25.6)	13.8 (10.0;18.3)	8.9 (5.5;12.4)	2.4 (0.6;4.6)

Legend: TXA, tranexamic acid.

## Data Availability

Not applicable.

## Appendix A

**Table A1:** Formula used for the calculation of the anthropometric and renal characteristics.

Parameters	Formula
ABW, adjusted body weight, kg	ABW= IW +0.4×(BW−IW)
BMI, body mass index, kg/m^2^	$\mathrm{BMI}=\frac{\mathrm{BW}}{\mathrm{Height}}$
LBW, lean body weight, kg	LBW=9270× BW/ [8780+(244 ∗ BMI) ]
IW, ideal weight, kg Devine et al., 1974, height in cm.	45.4+0.89×(Height (cm)−152.4)
BSA, body surface area, m^2^ Dubois and Dubois [24], height in m.	$0.20247\times {\mathrm{Height}}^{0.725}\times {\mathrm{BW}}^{0.425}$
eClcr, estimated creatinine clearance, mL/min Cockroft–Gault formula [25] SCr for serum creatinine in mg/dL.	$\frac{0.85\times \left(140-\mathrm{Age}\right)\times \mathrm{BW}}{\mathrm{SCr}\times 72}$
eGFR, estimated glomerular filtration rate, mL/min/1.73 m^2^, CKD-EPI formula [26] SCr for serum creatinine in mg/dL.	$\left[144\times {\left(\frac{\mathrm{SCr}}{0.7}\right)}^{-0.329}\times 0.993^{\mathrm{Age}}\right]\times \frac{\mathrm{BSA}}{1.73}$
eGFR, estimated glomerular filtration rate, mL/min/1.73 m^2^, MDRD formula [27] SCr for serum creatinine in mg/dL.	$\left[0.742\times 175\times {\mathrm{SCr}}^{-1.154}\times {\mathrm{Age}}^{-0.203}\right]\times \frac{\mathrm{BSA}}{1.73}$

## Appendix C

**Table A2:** Baseline characteristics of the patients who received a rescue dose (n = 8).

Patient	Age	BW	BWbef	eClcr	Blood Loss at T0	Inclusion Group	Time of Rescue Dose (Dose)	Additional Blood Loss at the Time of Second Dose	TXA Amount Measured in the Total Urine at T360
Units	-	kg	kg	mL·min^−1^	mL	-	Min	mL	mg
01-1-049	33	73	60	151.6	1019	TXA1g	117 (1 g)	802	1144
01-1-065	40	74	63	148.8	850	TXA1g	236 (1 g) and 300 (1 g)	1620	1058
01-1-070	36	66	53	72.3	1200	TXA1g	87 (1 g)	1175	1199
01-1-099	37	85	75	182.4	1308	TXA1g	36 (1 g)	345	1206
01-1-108	42	78	65	125.3	1275	TXA0.5g	72 (0.5 g)	695	1303
01-1-119	33	86	68	95.4	1020	TXA1g	50 (1 g)	1870	344
01-1-134	31	70	50	91.9	1720	TXA1g	153 (1 g)	2020	105
01-1-151	35	115	99	204.5	1900	TXA1g	35 (1 g)	600	648

Legend: BW, body weight; BWbef, body weight before pregnancy; TXA, tranexamic acid; T0, inclusion time; T360, 360 min after first TXA injection.

**Table A3:** Estimated PK parameters of the patients who received a rescue dose (n = 8).

Patient	CL	V1	V2	Q	Purine
Units	L·min^−1^	L	L	L·min^−1^	-
01-1-049	0.16	29.37	0.32	9.5	0.61
01-1-065	0.16	8.08	0.38	9.58	0.39
01-1-070	0.11	16.79	0.27	10.01	0.51
01-1-099	0.12	6.43	0.068	9.4	0.46
01-1-108	0.16	9.87	0.39	8.98	0.77
01-1-119	0.086	14.62	0.46	9.64	0.31
01-1-134	0.11	8.21	0.38	9.64	0.36
01-1-151	0.19	25.34	0.31	9.61	0.53
